# Model-Based Geostatistical Methods Enable Efficient Design and Analysis of Prevalence Surveys for Soil-Transmitted Helminth Infection and Other Neglected Tropical Diseases

**DOI:** 10.1093/cid/ciab192

**Published:** 2021-06-14

**Authors:** Olatunji Johnson, Claudio Fronterre, Benjamin Amoah, Antonio Montresor, Emanuele Giorgi, Nicholas Midzi, Masceline Jenipher Mutsaka-Makuvaza, Ibrahim Kargbo-Labor, Mary H Hodges, Yaobi Zhang, Collins Okoyo, Charles Mwandawiro, Mark Minnery, Peter J Diggle

**Affiliations:** 1 Centre for Health Informatics, Computing, and Statistics, Lancaster Medical School, Lancaster University, Lancaster, United Kingdom; 2 Department of Control of Neglected Tropical Diseases, World Health Organization , Geneva, Switzerland; 3 National Institute of Health Research, Ministry of Health and Child Care, Zimbabwe; 4 Neglected Tropical Disease Program, Ministry of Health and Sanitation, Freetown, Sierra Leone; 5 Helen Keller International, Regional Office for Africa, Dakar, Senegal; 6 Eastern and Southern Africa Centre of International Parasite Control, Kenya Medical Research Institute, Nairobi, Kenya; 7 School of Mathematics, College of Biological and Physical Sciences, University of Nairobi, Nairobi, Kenya; 8 Deworm the World, Evidence Action, Washington, District of Columbia, USA; 9 Health Data Research UK, London, United Kingdom

**Keywords:** control of neglected tropical diseases, geospatial analysis, impact survey, model-based geostatistics, prevalence survey, soil-transmitted helminth infection

## Abstract

Maps of the geographical variation in prevalence play an important role in large-scale programs for the control of neglected tropical diseases. Precontrol mapping is needed to establish the appropriate control intervention in each area of the country in question. Mapping is also needed postintervention to measure the success of control efforts. In the absence of comprehensive disease registries, mapping efforts can be informed by 2 kinds of data: empirical estimates of local prevalence obtained by testing individuals from a sample of communities within the geographical region of interest, and digital images of environmental factors that are predictive of local prevalence. In this article, we focus on the design and analysis of impact surveys, that is, prevalence surveys that are conducted postintervention with the aim of informing decisions on what further intervention, if any, is needed to achieve elimination of the disease as a public health problem. We show that geospatial statistical methods enable prevalence surveys to be designed and analyzed as efficiently as possible so as to make best use of hard-won field data. We use 3 case studies based on data from soil-transmitted helminth impact surveys in Kenya, Sierra Leone, and Zimbabwe to compare the predictive performance of model-based geostatistics with methods described in current World Health Organization (WHO) guidelines. In all 3 cases, we find that model-based geostatistics substantially outperforms the current WHO guidelines, delivering improved precision for reduced field-sampling effort. We argue from experience that similar improvements will hold for prevalence mapping of other neglected tropical diseases.

Soil-transmitted helminths (STHs) are a group of intestinal nematodes mainly composed of *Ascaris lumbricoides* (roundworms), *Trichuris trichiura* (whipworms), and *Necator americanus* and *Ancylostoma duodenale* (hookworms). Each of the 4 STH species has distinct characteristics, but control programs generally consider them as a single group because of their similar transmission dynamics, diagnosis, control, and prevention measures [1].

Infection with STHs affects both children and adults. It can lead to iron deficiency anemia, protein energy malnutrition, prolapsed rectum, and stunted growth. Severe cases can lead to intestinal obstructions and gangrene [2].

The STH parasites are transmitted to the human host through soil contaminated with human faeces containing the parasites. Repeated mass drug administration (MDA) with albendazole or mebendazole is used to control morbidity. Periodical surveys are necessary to monitor the impact of the intervention and adapt the control measures to the changing epidemiological situation [3].

World Health Organization (WHO) guidelines [4] specify that the prevalence of STHs in a designated geographical area should be estimated as the observed proportion of positive test results. The value of this estimate then determines whether MDA should continue and, if so, at what frequency [4]. This estimate is almost always inefficient and often substantially so, because it takes no account of geographical variation in risk within the area of interest.

The term *geostatistics* refers to a collection of statistical methods for learning about a spatially continuous phenomenon of scientific interest that varies over a designated geographical region, using data collected from a finite set of survey locations. Diggle et al [5] coined the term *model-based geostatistics* to mean the application of general principles of statistical method to geostatistical problems. These general principles include the following: design the data collection to yield data that are as informative as possible about the questions of scientific interest, subject to practical constraints; formulate a model that is as simple as possible while being compatible with both the data and existing scientific knowledge; use efficient statistical methods to ensure that predictions are as precise as possible.

Our aim in this study is to demonstrate the advantages of using model-based geostatistics to design and analyze prevalence surveys in low-resource settings. Our particular focus is on the design and analysis of impact surveys, that is, prevalence surveys that are conducted postintervention with the aim of informing decisions on what further intervention, if any, is needed to achieve elimination of the disease as a public health problem.

We use 3 case studies based on data from STH impact surveys in Kenya, Sierra Leone, and Zimbabwe to compare the predictive performance of model-based geostatistics with methods described in current WHO guidelines [4]. In all 3 cases, we find that model-based geostatistics substantially outperforms the current WHO guidelines, delivering improved precision for reduced field-sampling effort. We also argue from experience that similar improvements will hold for prevalence mapping of other neglected tropical diseases or, more generally, to any setting in which geographical variation in prevalence exhibits spatial correlation.

## METHODS

### Design of STH Prevalence Surveys

Monitoring is an integral component of any STH control program. Its purpose is to evaluate an implemented intervention and assess its impact. Surveys are usually conducted in schools or sentinel sites within a designated study region on the tacit assumption that data on prevalence in the surveyed schools will provide sufficient information to evaluate the program’s progress in the entire area. Surveys are usually targeted at school-aged children (SAC), among whom prevalence of roundworm and whipworm infections is typically higher than in pre-SAC and adults.

**Figure 1.**
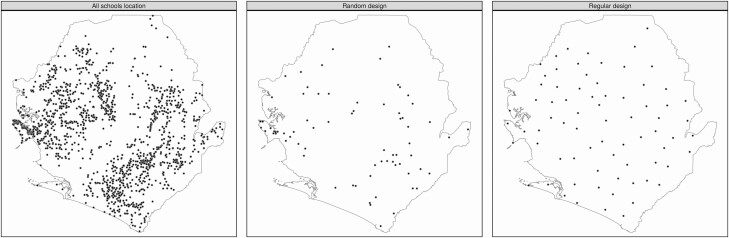
Locations of the 1559 candidate primary schools in Sierra Leone (left panel). Spatially random and spatial regulated designs for 73 sampled schools (middle and right panels, respectively). The spatially regulated design imposes a minimum distance of 20 km between any 2 sampled schools.

The main elements of an STH survey design are (1) the number of schools to sample; (2) the number of children to test in every school; and (3) the locations of the sampled schools. Current guidelines include a recommendation to sample 50 children per school to enable field researchers to survey a school in a single visit. The number of schools surveyed will be limited by the available budget. With regard to the locations of sampled schools, a random design (Figure 1) avoids any possibility of bias, but in the current geographical setting can result in sampling near-neighboring schools that partially duplicate each other’s information if prevalence exhibits smooth spatial variation over the study region. A way to counter this is to aim for a more even spatial distribution of sampled schools. However, if done subjectively, this risks introducing bias.

To achieve an even spatial distribution of sampled schools while avoiding subjective bias, we recommend the use of a spatially regulated sampling design (Figure 1).

This class of designs uses a constrained randomization that imposes a minimum distance between any 2 sampled locations and usually leads to better predictive performance [6].

Purposeful sampling, whereby schools are selected according to expert knowledge of the population and the distribution of the disease, also introduces bias. An objective way to take advantage of expert knowledge of this kind is to prestratify the study region, in which case we recommend spatially regulated sampling within each stratum.

Whatever design is used, it is advantageous to identify spatially varying environmental factors that are known, or likely, to be associated with spatial variation in prevalence and are available in the form of digital images covering the study region. Variables of this kind should be considered for inclusion as explanatory variables in the analysis of the survey data so as to improve predictive precision.

An implementation unit (IU) is a geographical area over which a particular treatment strategy will be applied; this could be a district, county, province, or whole country. After 5 or 6 years of MDA, it is expected that disease prevalence will be lowered to the point at which a reduction in the frequency of subsequent rounds of MDA can be considered. According to the WHO STH decision tree [4], after 5 years of twice-yearly application, the MDA regimen should continue according to a set of endemicity classes defined by prevalence thresholds as follows: suspend MDA if STH prevalence is less than 2%; conduct MDA every 2 years if STH prevalence is between 2% and 10%; annually if between 10% and 20%; twice yearly if between 20% and 50%; and thrice yearly if greater than 50%.

### Analyzing Prevalence Survey Data According to WHO Guidelines

According to WHO guidelines [4], the IU-level prevalence should be estimated by the formula


$$prevalence\text{ = }\frac{Number\;of\;STH\;cases}{Number\;of\;individuals\;tested}$$ (1)

This estimate is unbiased, albeit inefficient, provided that participants (schools and children) are randomly chosen from the general population. Also according to WHO guidelines, the estimate given in equation (1) should be used to classify each IU into the endemicity classes listed above, with no consideration given to its associated degree of uncertainty.

### Model-Based Geostatistical Analysis of Prevalence Survey Data

Diggle and Giorgi [7] give a complete account of the model-based geostatistical approach to the analysis of georeferenced health outcome data. A short outline follows.

Our outcome of interest is the prevalence of any STH infection. We write *P*(*x*) for the true prevalence at any location *x*. The data collected at each sampled location *x* include the number, *n*(*x*), of individuals whose disease status we ascertain using a suitable test instrument, and the number, *Y*(*x*), who return a positive test result. The estimate of *P*(*x*) implied by the WHO guidelines is the observed proportion of test-positive results, *q*(*x*) = *y* (*x*)/*n* (*x*), but this is of limited value for 2 reasons. First, it tells us nothing about the prevalence anywhere other than at *x*; second, except in very rare circumstances it is not even the best estimate of *P* (*x*) itself.

One way to obtain a better estimate is to collect data on measurements of 1 or more explanatory variables, *d*(*x*), attributes of *x* that we believe may be associated with *P*(*x*), and to fit a regression model, typically a logistic regression [8], to the complete data set. If values of *d* (*x*) are available at unsampled locations, for example as a set of digital images over the designated region, this addresses the first limitation of the naive estimate, *q* (*x*). But we can do better still by recognizing that in most cases, the available set of explanatory variables will not completely explain the spatial variation in prevalence. We therefore extend the logistic regression model by including an unobserved stochastic process, *S*(*x*), to represent the variation in *P*(*x*) that is not explained by *d* (*x*). The resulting geostatistical logistic model assumes that


log(P(x)/(1−P(x)))=d(x)'β+S(x)+Z

and that, given *P*(*x*), the observed test-positive counts, *y*(*x*), are independent and binomially distributed with probabilities *P*(*x*) and denominators *n*(*x*).

In this model, the essential feature of *S*(*x*) is that its values at different locations, *x* and *x*′ say, are correlated to an extent that depends on the distance between *x* and *x*′ in a manner that we can estimate from the data along with the regression parameters *β*. For reasons of statistical efficiency and probity, we advocate likelihood-based methods (maximum likelihood or Bayesian) to estimate the model parameters, and probabilistic methods to predict scientifically interesting features of the unobserved prevalence surface *P*(*x*).

### A Model-Based Decision Algorithm

We propose a probabilistic classification algorithm for IUs that proceeds as follows:

1. Fit to the available data a geostatistical logistic model including any available covariates that can help to explain the spatial variability in infection.
2. For each IU, draw samples from the predictive distribution of the IU-wide population-weighted prevalence.
3. Calculate the predictive probability of belonging to each of the 5 endemicity classes and assign to the IU the endemicity class with the highest probability.

### Case Studies

We have conducted simulations based on STH prevalence data collected in Zimbabwe [9], Kenya [10], and Sierra Leone [11] with 2 objectives: to evaluate the performance of our model-based decision algorithm under different sampling designs, and to compare the results with the performance of the WHO guideline in conjunction with random sampling of school locations. All 3 countries had undergone 4 or more rounds of MDA prior to data collection. All surveys targeted SAC and assessed infection by the Kato-Katz method [12] using slides prepared from a stool specimen collected from each child on the day of the survey (1 slide per person in Sierra Leone, 2 slides per person in Kenya and Zimbabwe). The objectives of each prevalence survey were to measure the impact of PC interventions on STH prevalence, to assess the endemicity level of each IU, and to decide the treatment regimen for subsequent years.

We created 56 candidate survey designs by varying the 3 key design elements as follows:

1. Number of schools to sample. We sampled a fraction, ranging between 40% and 100%, of the number of schools originally sampled in each country.
2. Number of children per school. We set this number at either 30, 50, 70, or 100.
3. Locations of the sampled schools. We compared random and spatially regulated designs, in each case without prestratification.

The true prevalence surface, P(x) is unknown. We therefore fitted a geostatistical model to the complete data set from each country, including various environmental characteristics as explanatory variables, as reported in the Results. From the fitted model we calculated the predicted prevalence surface and the corresponding endemicity class of each IU. We used the resulting classification as the benchmark relative to which we evaluated the performance of each candidate design, using as performance criterion the proportion of IUs correctly classified.

For each of the 56 candidate sampling designs, we replicated the following procedure 1000 times.

1. Simulate a synthetic data set from the benchmark prevalence surface.
2. Fit a geostatistical logistic model to the synthetic data set and assign to each IU an endemicity class using both the WHO guideline and the model-based decision algorithm.
3. Calculate the proportion of correctly classified IUs.

We then took as our measure of the performance of each candidate design the average, over all 1000 replicates, of the proportion of correctly classified IUs.

## RESULTS

Table 1 shows a summary of the survey data used for our 3 case studies. At the time of sampling, Zimbabwe had a much lower average prevalence than either Sierra Leone and Kenya. The 3 data sets also vary substantially in size.

**Table 1.** Summary Statistics for Soil-Transmitted Helminth Surveys Conducted in Kenya, Sierra Leone, and Zimbabwe

Country	Survey Year	MDA Rounds	No. of Schools	No. of Cases Examined		Cases Detected		Prevalence		
				Total	Mean	Total	Mean	Minimum	Mean	Maximum
Kenya	2017	4	172	17 936	104	2712	16	0	15.0	67.6
Sierra Leone	2016	6	73	3632	50	663	9	0	18.3	53.1
Zimbabwe	2018	6	336	12 537	37	96	0	0	0.8	25.0

Abbreviation: MDA, mass drug administration.

**Figure 2.**
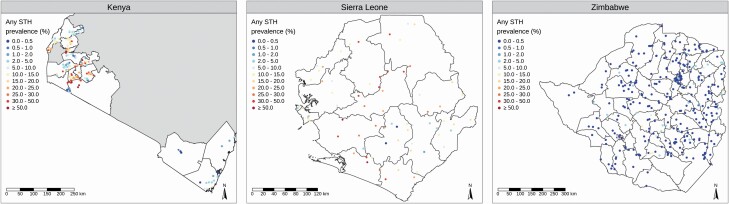
Empirical prevalence of any soil-transmitted helminth (STH) infection at each sampled location in Kenya, Sierra Leone, and Zimbabwe.

**Figure 3.**
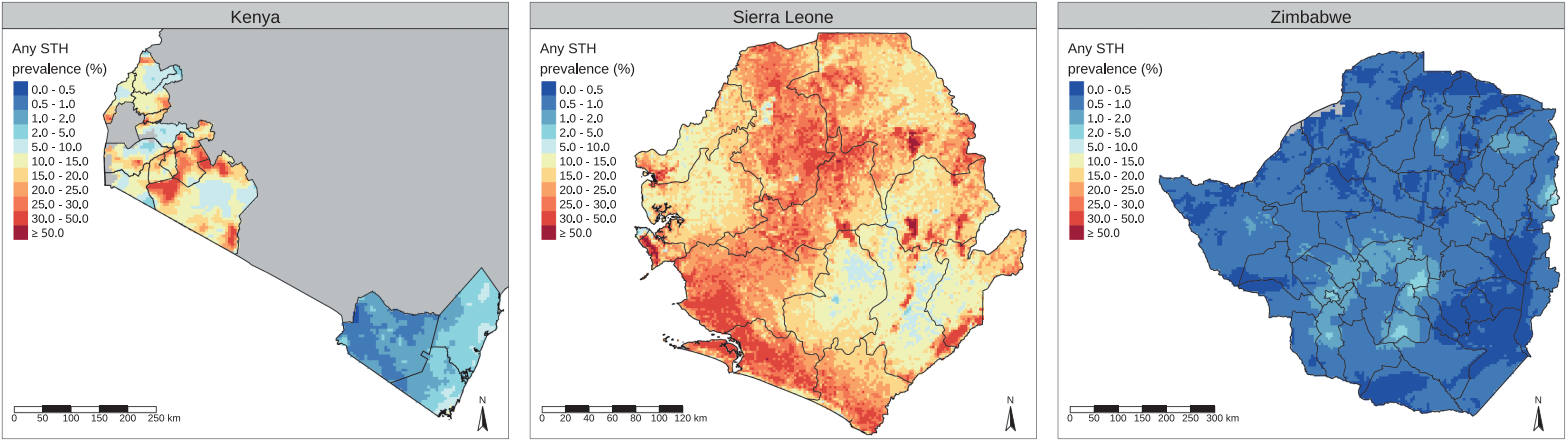
Predicted prevalence surfaces for Kenya, Sierra Leone, and Zimbabwe. Abbreviation: STH, soil-transmitted helminth.

Figure 2 shows, for each country, the empirical STH prevalence at each sampled location. We fitted a geostatistical model to the data from each country as described above (“Model-Based Geostatistical Analysis of Prevalence Survey Data”), including in each case selecting covariates from a set of spatially varying environmental factors that are known to be potential drivers of STH infection (Supplementary Material 3). Figure 3 shows the resulting predicted prevalence surfaces that, as noted earlier, provide the benchmark for our performance comparisons.

### Kenya

The data for the Kenya case study were collected in an impact assessment survey of 17 936 children in 172 schools. The survey was conducted in 2017 after 4 rounds of MDA [10]. Our fitted geostatistical model included 3 environmental covariates: enhanced vegetation index, day and night land surface temperature, and soil acidity. Because the surveyed schools were located in 2 widely separate regions of Kenya with different historical levels of STH prevalence, the model also included region as a 2-level factor (Supplementary Material 3).

**Figure 4.**
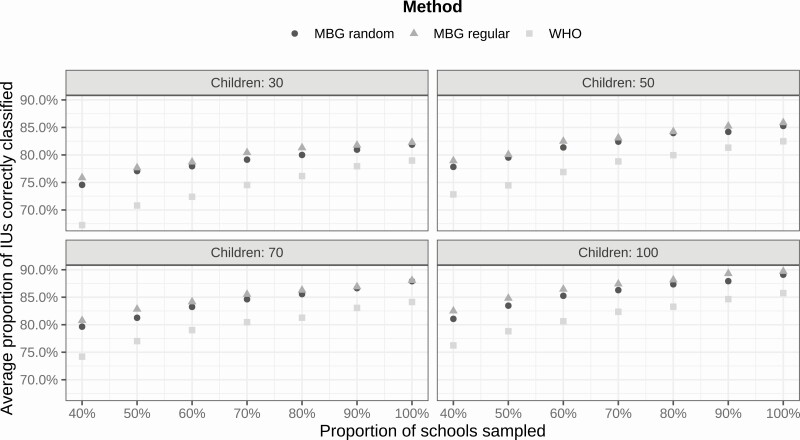
Performance of different sampling designs in the Kenya case study. Average proportion of correctly classified implementation units by number of schools (as percentage of number used in original survey), number of children per school (30, 50, 70, or 100), and spatial sampling design (spatially random or spatially regulated). Abbreviations: IU, implementation unit; MBG, model-based geostatistics; WHO, World Health Organization.

Figure 4 shows the results. Using model-based geostatistics, spatially regulated sampling slightly outperforms spatially random sampling, and both comfortably outperform the WHO guideline.

### Sierra Leone

The data for the Sierra Leone case study were collected in an impact assessment survey of 3632 children in 73 schools. The survey was conducted in 2016 after 6 rounds of MDA [11]. Our fitted geostatistical model included 2 environmental covariates: soil sand content and soil pH (Supplementary Material 3).

**Figure 5.**
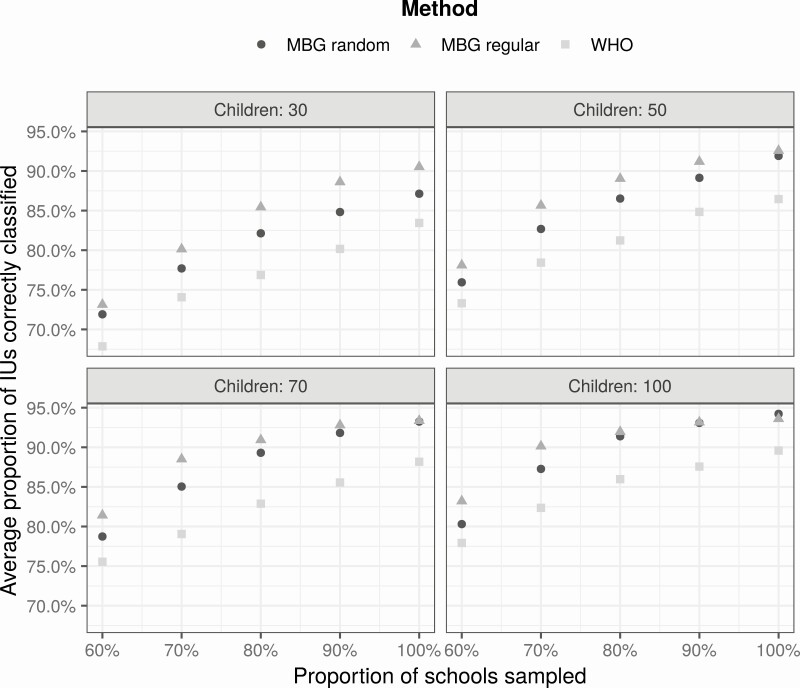
Performance of different sampling designs in the Sierra Leone case study. Average proportion of correctly classified implementation units by number of schools (as percentage of number used in original survey), number of children per school (30, 50, 70, or 100), and spatial sampling design (spatially random or spatially regulated). Abbreviations: IU, implementation unit; MBG, model-based geostatistics; WHO, World Health Organization.

Figure 5 shows the results. Both model-based geostatistical analyses outperform the WHO guideline. Also, spatially regulated sampling outperforms spatially random sampling by a greater margin than in Kenya.

### Zimbabwe

The data for the Zimbabwe case study are from an impact assessment survey of 12 537 children in 336 schools. The survey was conducted between September and December 2018 after 6 years of MDA [9]. Our fitted geostatistical model included 3 environmental covariates: night-light emission, soil moisture, and soil pH (Supplementary Material 3).

**Figure 6.**
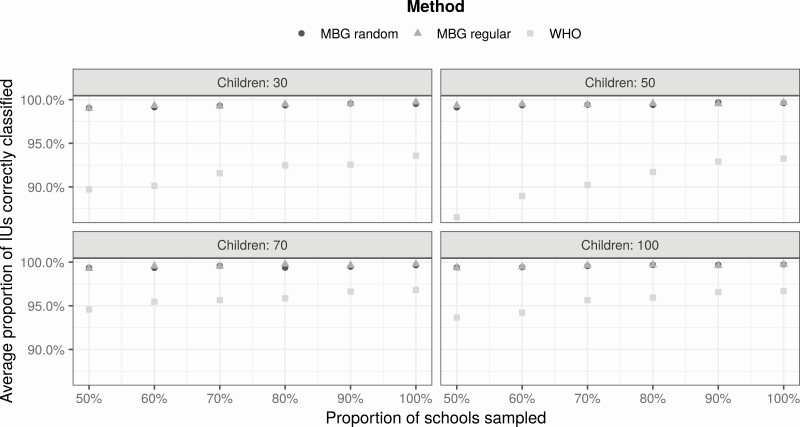
Performance of different sampling designs in the Zimbabwe case study. Average proportion of correctly classified implementation units by number of schools (as percentage of number used in original survey), number of children per school (30, 50, 70, or 100), and spatial sampling design (spatially random or spatially regulated). Abbreviations: IU, implementation unit; MBG, model-based geostatistics; WHO, World Health Organization.

Figure 6 shows the results. Using model-based geostatistics, the performances of spatially random and spatially regulated sampling designs are almost identical, and substantially better than the performance obtained using the WHO guideline.

## Discussion

Our first and strongest finding is that in all cases a model-based geostatistical analysis, whether used in conjunction with a spatially random or a spatially regulated design, substantially outperforms the WHO guideline with respect to correct classification of the endemicity class of an IU. Adoption of the model-based geostatistical approach would therefore enable future prevalence studies to achieve improved precision with smaller sample sizes than have been used hitherto. In practice, the size of any prevalence survey is constrained by the available budget. The optimum balance between the number of schools and the number of children per school sampled then depends on the relative costs of travel to schools and the processing of test results.

Our second finding is that a spatially regulated sampling design performs at least as well as, and sometimes substantially better than, a spatially random design. This was established theoretically in the case of unrestricted sampling locations in a 1960 Swedish PhD thesis by Matérn [13]. In the current context, sampling locations are restricted to the locations of schools and, as our case studies demonstrate, this can limit the gains in efficiency that accrue from spatially regulated sampling by comparison with spatially random sampling.

Purposeful sampling involves the deliberate favoring of some survey locations because of their known or suspected characteristics; for example, in monitoring for postelimination recrudescence, sampling in historical areas of high prevalence has an obvious rationale. However, for country-wide assessment of prevalence, this brings an equally obvious risk of bias. A better strategy is to prestratify the study area, use a randomized (spatially random or regulated) sampling design within each stratum, and to include stratum as a factor in the fitted geostatistical model. When data from preintervention prevalence surveys are available, these can be used to design a stratified impact survey in which areas of historically low prevalence can be undersampled, resulting in a more efficient use of limited resources.

When the goal of a survey is to predict, rather to understand the causes of, spatial variation in prevalence, covariate effects are not of direct interest. But they are still important, because their inclusion can improve precision by transferring what would otherwise be unexplained, stochastic variation in prevalence to explained, systematic variation. Judgments on which covariates to include should, however, be made with care, because in any particular setting, environmental variables that are known to be generally associated with prevalence may nevertheless make negligible contributions to variation in prevalence within the study area. Inclusion of such covariates can lead to reduced, rather than enhanced, precision.

Other than determining a relevant set of candidate environmental covariates, the specific disease context has no bearing on the model-based geostatistics approach to prevalence survey design and analysis. We have used it in, among other settings, studies of the spatial variation in prevalence of loaiasis [14, 15], onchocerciasis [16], malaria [17], snakebite [18], and lymphatic filariasis [19]. Based on this wide experience, we contend that the advantages of a model-based geostatistical approach over traditional approaches to survey design and analysis are not limited to the STH setting of the current study but will hold for any setting in which geographical variation in prevalence exhibits spatial correlation.

In conclusion, this study adds to the evidence in support of a novel approach to reduce the cost of monitoring and evaluation of control activities for STH and other neglected tropical diseases. However, an obstacle to the widespread adoption of this approach is its need for additional expertise in statistical analysis. To this end, WHO, in collaboration with the Centre for Health Informatics, Computing, and Statistics (CHICAS) at Lancaster University, is engaged in providing training and computational tools to facilitate the collection and geostatistical analysis of epidemiological data in endemic countries.

## Supplementary Data

Supplementary materials are available at *Clinical Infectious Diseases* online. Consisting of data provided by the authors to benefit the reader, the posted materials are not copyedited and are the sole responsibility of the authors, so questions or comments should be addressed to the corresponding author.

ciab192_suppl_Supplementary-Material

## Notes

***Supplement sponsorship.*** This article appears as part of the supplement “Sustainable control of neglected tropical diseases – surveillance and diagnostics”, sponsored by the NTD Modelling Consortium.

***Disclaimer.*** The funders had no role in the study design, data collection and analysis, preparation of the manuscript, or decision to submit the manuscript for publication.

***Financial support.*** This work was supported by the Bill & Melinda Gates Foundation through a grant to the Neglected Tropical Diseases Modelling Consortium (“Quantitative analysis and modelling on targets and strategies for alleviating the burden of priority neglected tropical diseases”); the United States Agency for International Development (USAID) through a grant to Helen Keller International (cooperative agreement number GHS-A-00-06-00006-00) with the End NTDs in Africa project managed by Family Health International 360; the End Fund, GiveWell, and Epic Foundation via Evidence Action through partial funding of the Kenya case study; and the Expanded Special Project for Elimination of Neglected Tropical Diseases (ESPEN), the World Health Organization, and Helen Keller International through funding of the baseline and impact assessment surveys in Zimbabwe.

***Potential conflicts of interest.*** M. M. reports grants from GiveWell and the Epic Foundation, outside the submitted work. All other authors report no potential conflicts of interest.

## References

[1] Mascarini-Serra L . Prevention of soil-transmitted helminth infection. J Glob Infect Dis 2011; 3:175–82. 21731306 10.4103/0974-777X.81696 PMC3125032

[2] Parija SC , Chidambaram M, Mandal J. Epidemiology and clinical features of soil-transmitted helminths. Trop Parasitol 2017; 7:81–5. 29114484 10.4103/tp.TP_27_17 PMC5652059

[3] Montresor A , Mupfasoni D, Mikhailov A, et al. The global progress of soil-transmitted helminthiases control in 2020 and World Health Organization targets for 2030. PLoS Negl Trop Dis 2020; 14:e0008505. 32776942 10.1371/journal.pntd.0008505 PMC7446869

[4] World Health Organization. Helminth control in school-age children: a guide for managers of control programs. Geneva, Switzerland: WHO, 2011.

[5] Diggle PJ , Moyeed RA, Tawn JA. Model-based geostatistics (with Discussion). Appl Stat 1998; 47:299–350.

[6] Chipeta MG , Terlouw DJ, Phiri KS, et al. Inhibitory geostatistical designs for spatial prediction taking account of uncertain covariance structure. Environmetrics 2017; 28. doi:10.1002/env.2425.

[7] Diggle PJ , Giorgi E. Model-based geostatistics for global public health: methods and applications. Boca Raton, FL: CRC Press, 2019.

[8] Hosmer DS , Lemeshow S, Sturdivant RX. Applied logistic regression. 3rd ed. Hoboken, NJ: Wiley, 2013.

[9] Midzi N , Montresor A, Mutsaka-Makuvaza MJ, et al. Elimination of STH morbidity in Zimbabwe: results of 6 years of deworming intervention for school-age children. PLoS Negl Trop Dis 2020; 14:e0008739. 33095760 10.1371/journal.pntd.0008739 PMC7641467

[10] Mwandawiro C , Okoyo C, Kihara J, et al. Results of a national school-based deworming programme on soil-transmitted helminths infections and schistosomiasis in Kenya: 2012-2017. Parasit Vectors 2019; 12:76. 30732642 10.1186/s13071-019-3322-1 PMC6367841

[11] Bah YM , Bah MS, Paye J, et al. Soil-transmitted helminth infection in school age children in Sierra Leone after a decade of preventive chemotherapy interventions. Infect Dis Poverty 2019; 8:41. 31262367 10.1186/s40249-019-0553-5 PMC6604471

[12] World Health Organization. Bench aids for the diagnosis of intestinal parasites. Geneva, Switzerland: WHO, 2019.

[13] Matérn B. Spatial variation. Stockholm: Meddelanden fran Statens Skogsforsknings Institut, 1960.

[14] Diggle PJ , Thomson MC, Christensen OF, et al. Spatial modelling and the prediction of *Loa loa* risk: decision making under uncertainty. Ann Trop Med Parasitol 2007; 101:499–509. 17716433 10.1179/136485913X13789813917463

[15] Zouré HG , Wanji S, Noma M, et al. The geographic distribution of *Loa loa* in Africa: results of large-scale implementation of the rapid assessment procedure for loiasis (RAPLOA). PLoS Negl Trop Dis 2011; 5:e1210. 21738809 10.1371/journal.pntd.0001210 PMC3125145

[16] Zouré HGM , Noma M, Tekle AH, et al. The geographic distribution of onchocerciasis in the 20 participating countries of the African Programme for Onchocerciasis Control: (2) pre-control endemicity levels and estimated number infected. Parasit Vectors 2014; 7:326. 25053392 10.1186/1756-3305-7-326 PMC4222889

[17] Kabaghe A , Chipeta MG, McCann RS, et al. Adaptive geostatistical sampling enables efficient identification of malaria hotspots in repeated cross-sectional surveys in rural Malawi. PLoS One 2016; 12:e0172266. 10.1371/journal.pone.0172266 PMC5308819 28196105

[18] Ediriweera DS , Kasturiratne A, Pathmeswaran A, et al. Mapping the risk of snakebite in Sri Lanka—a national survey with geospatial analysis. PLoS Negl Trop Dis 2016; 10:e0004813. 27391023 10.1371/journal.pntd.0004813 PMC4938527

[19] Fronterre C , Amoah B, Giorgi E, Stanton MC, Diggle PJ. Design and analysis of elimination surveys for neglected tropical diseases. J Infect Dis 2020; 221:554–60. 10.1093/infdis/jiz554 PMC7289555 31930383

