# Supplementary material for: Model-Based Geostatistical Methods Enable Efficient Design and Analysis of Prevalence Surveys for Soil-Transmitted Helminth Infection and Other Neglected Tropical Diseases
Source: Clin Infect Dis. 2021 Jun 14;72(Suppl 3):S172–9. doi: 10.1093/cid/ciab192 (PMC8201574; doi:10.1093/cid/ciab192)
Supplement: ciab192_suppl_Supplementary-Material [file ciab192_suppl_supplementary-material.docx]

**Supplementary Material for “Model-based Geostatistical Methods Enable Efficient Design and Analysis of Prevalence Surveys for Soil-Transmitted Helminth Infection and Other Neglected Tropical Diseases”**

Olatunji Johnson^a^, Claudio Fronterre^a^, Benjamin Amoah^a^ Antonio Montresor^b^, Emanuele Giorgi^a^, Nicholas Midzi^c^, Masceline Jenipher Mutsaka-Makuvaza^c^, Ibrahim Kargbo-Labor^d^,

Mary H. Hodges^e^, Yaobi Zhang^e^, Collins Okoyo^g,h^, Charles Mwandawiro^g^, Mark Minnery^i^ and

Peter J Diggle^a,f^

**S.1 The geostatistical model**

We denote by $P\left( x \right)$ the prevalence of STH at location $x$. Our model for the variation in $P\left( x \right)$throughout the region of interest is that

$log\left( {P\left( x \right)}/\left( 1-P\left( x \right) \right) \right)=d\left( x \right)'\beta+S\left( x \right)+Z.$ Eqn (1)

In Equation (1), $d\left( x \right)$ is a vector of covariates associated with regression coefficients β. This component of the model accounts for variation in prevalence that can be explained by measured characteristics of the location $x$; see Table S3. The terms $S\left( x \right)$ and $Z$ account for any remaining variation that cannot be explained by measured characteristics of $x$. The term $S\left( x \right)$is a spatially correlated Gaussian process with mean zero and covariance structure

$Cov\left( S\left( x \right),S\left( x' \right) \right)=\sigma^{2}\rho\left( u;\theta\right),$

where $u=\left\| x-x' \right\|$ is the Euclidean distance between $x$ and $x^{'},$ $\sigma^{2}$ is the variance and

$\rho\left( u;\phi\right)=\exp\left( -\frac{u}{\phi} \right)$

is the correlation between $S\left( x \right)$ and $S\left( x' \right)$. The term $Z$ in Equation (1) is a Normally distributed random variable with zero mean and variance $\tau^{2}$ that varies independently between locations; it accounts for variation in unmeasured characteristics of the sampled individuals that affect their personal exposure to STH.

We denote by $x_{1},\ldots,x_{n}$ the set of sampled locations. Conditional on $P\left( x_{i} \right)$, the numbers $Y_{i}$ of individuals who test positive out of $m_{i}$ sampled individuals at $x_{i}$ are independent binomially distributed random variables, with binomial probabilities $P\left( x_{i} \right)$ and denominators $m_{i}$.

**S.2 Parameter estimation**

We carry out parameter estimation using Monte Carlo Maximum Likelihood (MCML), implemented in PrevMap, an R package for analysing prevalence data, freely available from the Comprehensive R Archive Network (*www.r-project.org*).

Let $\eta_{i}=\log\left( {P\left( x \right)}/\left( 1-P\left( x \right) \right) \right)$. The joint conditional density of $Y=Y_{1},\ldots,Y_{n}$is

$$f\left( y|\eta\right)=\prod_{i=1}^{n} f\left( y_{i}|\eta_{i} \right).$$

The likelihood function for the set of model parameters $\psi$ is obtained by integrating out the random components$S\left( x_{i} \right)$and$Z_{i}$ from $\eta_{i}$, hence

$L\left( \psi\right)=\int_{R^{n}} f\left( y|\eta\right) f\left( \eta;\psi\right)d\eta$, Eqn (2)

where$f\left( \eta;\psi\right)$is a multivariate Normal density.

To approximate the integral in Equation 2 we use a Markov Chain Monte Carlo (MCMC) algorithm to generate a sample $\eta_{\left( 1 \right)},...,\eta_{\left( N \right)}$ from the conditional distribution of $\eta$ given $y$ and approximate the likelihood as

$$L\left( \psi\right)\propto L_{N}\left( \psi\right)=\frac{1}{N}\frac{\sum_{j=1}^{N} f\left( \eta_{\left( j \right)};\psi\right)}{f\left( \eta_{\left( j \right)};\psi_{0} \right)},$$

Where $\psi_{0}$ is our best guess for the initial parameter values.

**Prediction**

Here, we use plug-in prediction, meaning that we use the Monte Carlo maximum likelihood parameter estimate $\hat{\psi}$ in place of the unknown $\psi$.

Our goal is to predict prevalence throughout the region of interest, $A$. We approximate this by a regular grid of points $x_{n+1},...,x_{n+q}$ that cover $A$. Our predictive target is the set of values

$\eta_{n+i}=d\left( x_{n+i} \right)'\beta+S\left( x_{n+i} \right)$ Eqn (3)

Note that Equation 3 excludes the term $Z$ in Equation 1, which relates to characteristics of the sampled individuals at a location rather than of the location itself.

The *predictive distribution* of $\eta^{*}=\left( \eta_{n+1},...,\eta_{n+q} \right)$ is its conditional distribution given $y$,

$$f\left( \eta^{*}|y \right)=\int_{R^{n}} f\left( \eta^{*}|\eta\right)f\left( \eta|y \right)d\eta,$$

where we have used the fact that $\eta^{*}$ and $y$ are conditionally independent given $\eta$. It follows that to generate a sample from the predictive distribution of $\eta^{*}$ we first sample from $f\left( \eta|y \right)$ and then from$f\left( \eta^{*}|\eta\right)$. A sample from the joint predictive distribution of prevalence throughout $A$ follows by direct transformation, using the formula

$$P\left( x_{n+i} \right)=\frac{\exp\left( \eta_{n+1}^{*} \right)}{1+\exp\left( \eta_{n+1}^{*} \right)}.$$

Our point prediction of prevalence at any location $x$ is the mean of the sampled values of $P\left( x \right)$. The predictive probability that prevalence lies within any stated range, say $c$ to $d$, is the proportion of sampled values that lie between $c$and$d$. The predictive distribution of prevalence at the implementation unit level, $P_{IU}$, is computed as a population-weighted average of the pixel level prevalence $P\left( x \right)$, hence

$$P_{IU}=\frac{\int P\left( x \right)w\left( x \right)dx}{\int w\left( x \right)dx}$$

where $w\left( x \right)$ is an estimate of the population at location $x$ obtained from WorldPop (*https://www.worldpop.org/*) and the integral is over the whole of the IU.

**S.3. Monte Carlo maximum likelihood estimates and corresponding 95% confidence intervals.**

| **Parameter** | ***Kenya*** | ***Sierra Leone*** | **Zimbabwe** |
| --- | --- | --- | --- |
| Intercept | -3.945  (-4.833, -3.058) | 17.310  (3.249, 31.371) | -1.677  (-3.650, -0.096) |
| EVI | 0.340  (0.046, 0.633) | NA | NA |
| LST day | -0.604  (-0.969, -0.239) | NA | NA |
| LST night | 0.647  (0.184, 1.11 ) | NA | -0.038  (-0.103, 0.027) |
| Soil acidity | -0.124  (-0.5, 0.252) | -0.314  (-0.553, -0.075) | -0.463  (-0.788, -0.137) |
| region NYANZA | 1.691  (0.599, 2.784) | NA | NA |
| region RIFT VALLEY | 2.584  (1.383, 3.784) | NA | NA |
| region WESTERN | 0.063  (0.035, 0.092) | NA | NA |
| Soil sand content | NA | -0.040  (-0.077, -0.003) | NA |
| soil moisture | NA | NA | -0.006  (-0.012 -0.001) |
| NLE | NA | NA | -0.035  (-0.106, -0.006) |
| $\sigma^{2}$ | 0.702  (0.426, 1.156) | 0.342  (0.193, 0.490) | 0.218  (0.036, 0.473) |
| $\phi$ | 11.564  (5.448, 24.543) | 35.193  (5.320, 65.707) | 10.203  (6.780, 17.186) |
| $\tau^{2}$ | 0.176  (0.029, 1.07) | NA | 0.148  (0.038, 0.234) |

Note: NA corresponds to a situation when the term is not included in the model.
